# HDL Size is More Accurate than HDL Cholesterol to Predict Carotid Subclinical Atherosclerosis in Individuals Classified as Low Cardiovascular Risk

**DOI:** 10.1371/journal.pone.0114212

**Published:** 2014-12-03

**Authors:** Eliane Soler Parra, Natalia Baratella Panzoldo, Vanessa Helena de Souza Zago, Daniel Zanetti Scherrer, Fernanda Alexandre, Jamal Bakkarat, Valeria Sutti Nunes, Edna Regina Nakandakare, Eder Carlos Rocha Quintão, Wilson Nadruz-Jr, Eliana Cotta de Faria, Andrei C. Sposito

**Affiliations:** 1 Department of Cardiology, Faculty of Medical Sciences, University of Campinas, Campinas, SP, Brazil; 2 Department of Clinical Pathology, Lipid Laboratory and Center for Medicine and Experimental Surgery, Faculty of Medical Sciences, University of Campinas, Campinas, SP, Brazil; 3 Lipid Laboratory, Faculty of Medical Sciences, University of São Paulo, São Paulo, SP, Brazil; University of Kansas Medical Center, United States of America

## Abstract

**Background:**

Misclassification of patients as low cardiovascular risk (LCR) remains a major concern and challenges the efficacy of traditional risk markers. Due to its strong association with cholesterol acceptor capacity, high-density lipoprotein (HDL) size has been appointed as a potential risk marker. Hence, we investigate whether HDL size improves the predictive value of HDL-cholesterol in the identification of carotid atherosclerotic burden in individuals stratified to be at LCR.

**Methods and Findings:**

284 individuals (40–75 years) classified as LCR by the current US guidelines were selected in a three-step procedure from primary care centers of the cities of Campinas and Americana, SP, Brazil. Apolipoprotein B-containing lipoproteins were precipitated by polyethylene glycol and HDL size was measured by dynamic light scattering (DLS) technique. Participants were classified in tertiles of HDL size (<7.57; 7.57–8.22; >8.22 nm). Carotid intima-media thickness (cIMT) <0.90 mm (80^th^ percentile) was determined by high resolution ultrasonography and multivariate ordinal regression models were used to assess the association between cIMT across HDL size and levels of lipid parameters. HDL-cholesterol was not associated with cIMT. In contrast, HDL size >8.22 nm was independently associated with low cIMT in either unadjusted and adjusted models for age, gender and Homeostasis Model Assessment 2 index for insulin sensitivity, ethnicity and body mass index (Odds ratio 0.23; 95% confidence interval 0.07–0.74, p = 0.013).

**Conclusion:**

The mean HDL size estimated with DLS constitutes a better predictor for subclinical carotid atherosclerosis than the conventional measurements of plasma HDL-cholesterol in individuals classified as LCR.

## Introduction

It is of particular concern that, depending of the risk score applied, up to 72% of patients admitted with ST-elevation would have originally been classified to be at low cardiovascular (CV) risk just prior to the event [1]. Such limitation of risk algorithms draws attention to the possible inconsistencies between a few of their risk markers and their true CV risk. In this context, the discriminatory power of plasma high-density lipoprotein (HDL) cholesterol has been shown to be highly heterogeneous among individuals and tends to be negligible among those with CV disease [2]. From a mechanistic point of view, this phenomenon is explained by an increase in the dysfunctional behavior of HDL that follows the exposition to CV risk factors. Still, in general, the inclusion of HDL cholesterol (HDL-C) in risk estimation is expected to improve the real risk assessment in only 2.2% [3]. Hence, it became evident that risk assessment would improve with the use of simple feasible markers of HDL function.

Cholesterol efflux capacity of HDL has been shown to be a step forward from plasma HDL-C in discriminating individuals with or without coronary or carotid atherosclerosis [4], [5]. Such improvement in prediction has been mainly attributed to phenotypic changes in HDL, which are not discernible from basic lipid profile assays. Studies using either native HDL or reconstituted HDL particles demonstrated that cholesterol efflux capacity is directly proportional to HDL size [6], [7]. As the diameter of HDL enlarges, changes occur in the conformation of the central region of the apolipoprotein (apo) A-I [6]. In turn, this leads to a greater HDL affinity for scavenger receptor class B type I (SRBI) and increased cell cholesterol efflux [6], [7].

Cholesterol efflux assessment, however, is an intricate, labor-intensive procedure that remains restricted to research laboratories. On the other hand, the assessment of HDL diameter may be obtained by straightforward and fast throughput technologies such as nuclear magnetic resonance (NMR) or dynamic light scattering (DLS) - the latter much less expensive and accessible in the clinical setting. In light of all of this, our main objective was to investigate whether HDL size assessment obtained from a simple feasible assay would improve the predictive value of HDL-C in the identification of individuals presenting subclinical atherosclerotic disease among those classified as low CV risk according to the most recent Atherosclerosis Cardiovascular Disease risk score (ASCVD) [8]. In addition, we investigated the main potential mechanisms that would justify differences in HDL size.

## Methods

### Subjects

**Figure 1:**
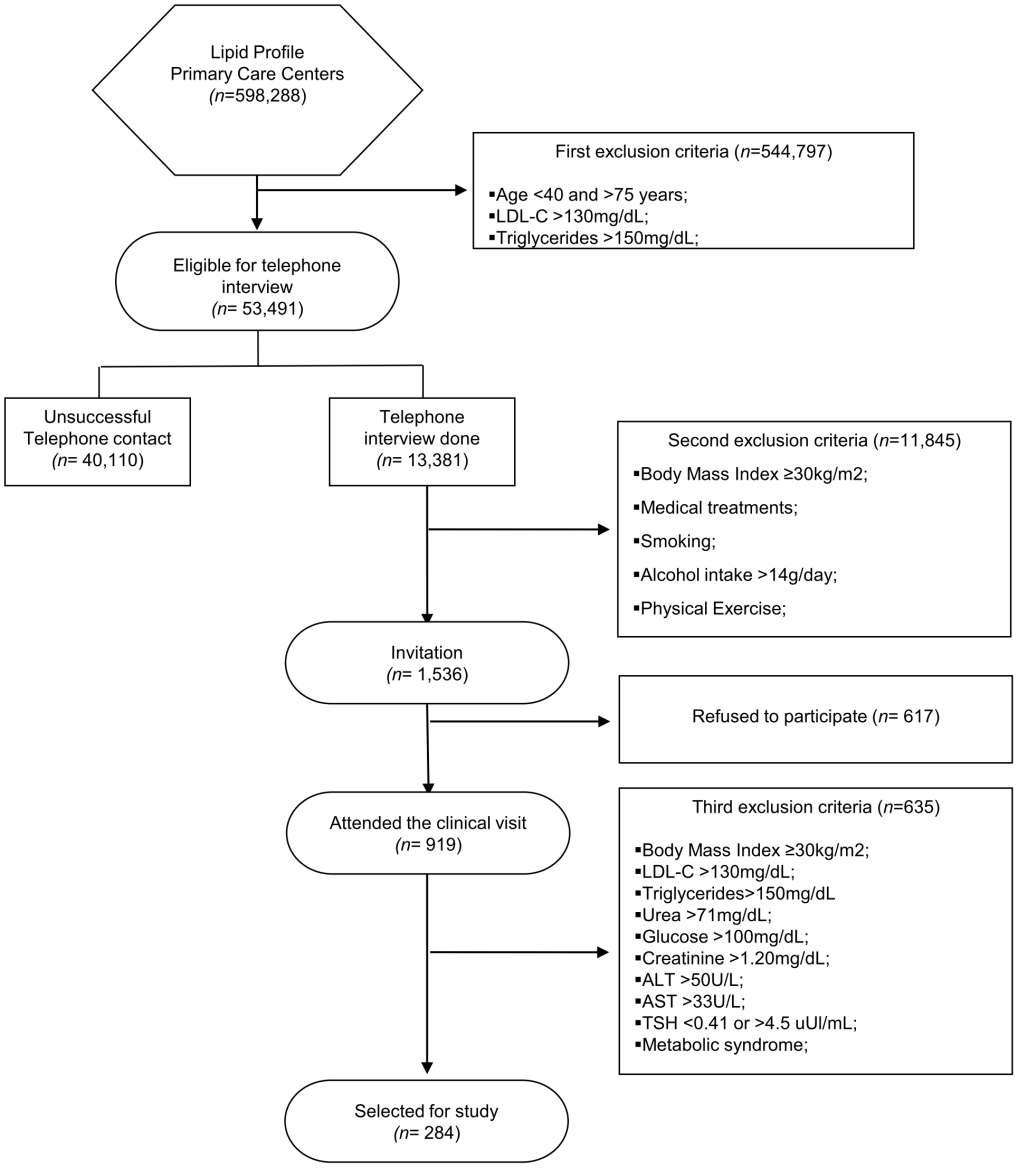
Flow-diagram of the study. ALT: alanine aminotransferase; AST: aspartate aminotransferase; THS: thyroid stimulating hormone.

Participants were selected in tree steps from a database of 598,288 lipid profiles of individuals who spontaneously sought governmental primary care centers for CV risk estimation between 2008 and 2011 in the cities of Campinas and Americana, SP, Brazil. Our goal was to select individuals aged 40 years or older at low CV risk and without regular use of lipid-lowering treatment or secondary causes for reduced HDL-C. In the first step, we selected medical reports from individuals with (i) low-density lipoprotein cholesterol (LDL-C) ≤130 mg/dL, (ii) triglycerides ≤150 mg/dL, and (iii) of both genders. In this phase, 53,491 individuals were considered eligible for telephone interview. We then excluded individuals who self-reported: (i) body mass index (BMI) ≥30 kg/m^2^, (ii) regular use of medical treatments, (iii) smoking habit, (iv) daily intake of alcohol >14 g or (v) intensive daily physical exercise. From 1,536 individuals who were selected and invited for in-person clinical evaluation and blood exams, 919 individuals attended the second step evaluation. During the second step, exclusions were made based of reassessment of BMI, LDL-C and triglycerides values as above reported as well as (i) urea >71 mg/dL, (ii) creatinine >1.20 mg/dL, (iii) glucose >100 mg/dL, (iv) alanine aminotransferase >50 U/L, (v) aspartate aminotransferase >33 U/L, (vi) thyroid stimulating hormone <0.41 or >4.5 uUI/mL, and (vii) metabolic syndrome as defined by the International Diabetes Federation (IDF) criteria. In this last step, 284 individuals who were considered eligible by the abovementioned criteria were enrolled. The flow diagram of this selection process is depicted in Figure 1.

### Ethics statement

The Ethics Committee in Medical Sciences of the University of Campinas approved this study (409/2010) and the study is registered at ClinicalTrials.Gov by the following identification NCT02106013. All volunteers signed an informed consent form before taking part in the study.

### Clinical and anthropometric data

Weight, height, BMI, waist and hip circumference, and systolic and diastolic blood pressure were obtained in duplicates. The ethnicity was self-reported and categorized as white or non-white. Estimated lipid accumulation product (LAP, for men =  (waist circumference - 65) × triglycerides) for women =  (waist circumference - 58) × triglycerides)) was used for estimating body lipid accumulation [9].

### Biochemical analysis

Blood samples were drawn after a 12-h fast and EDTA plasma was separated by centrifugation (4°C, 1000×*g*, 10 minutes) and stored at −80°C until analysis. Total cholesterol, triglycerides, HDL-C and glucose measurements were performed in an automated chemical analyzer Modular Analytics Evo (Roche Diagnostics, Burgess Hill, West Sussex, UK), using Roche Diagnostics reagents (Mannheim, Germany). LDL-C was calculated by Friedewald's equation. Apo A-I, apo B and lipoprotein (a) were determined by nephelometry in a BNII automated system and reagents from Dade-Behring (Marburg, Germany). C-reactive protein (CRP) was measured using the Tina-quant CRP (latex) high sensitivity assay (Roche Diagnostics, Mannheim, Germany) by immunoturbidimetry. Plasma insulin was determined by ELISA (Human Insulin ELISA kit, Millipore Corporation, MA, USA).

The Modification of Diet in Renal Disease (MDRD) equation estimated glomerular filtration rate (GFR). The Homeostasis Model Assessment 2 (HOMA2) Calculator version 2.2 was used to estimate β cell function (HOMA2B) and insulin sensitivity (HOMA2S) [10]. Cholesteryl ester transfer protein (CETP) [11] and phospholipid transfer protein (PLTP) activities in plasma were determined using radioassays with exogenous substrates and PLTP mass was measured by ELISA as previously describe [12]. PLTP specific activity was calculated as the ratio of PLTP activity and PLTP concentration. Paraoxonase-1 (PON-1) activity was measured using paraoxon (diethyl-*p*-nitrophenylphosphate, Sigma, St. Louis, MO, USA) as substrate [13]. A subgroup of 159 individuals were randomly selected and were analyzed the exogenous lecithin cholesterol acyltransferase (LCAT) activity (nmolCE/mL/h), performed using a recombinant HDL as substrate [14], additionally, LCAT endogenous activity (% cholesterol ester) was measured through the rate of esterification of ^14^C-free cholesterol by LCAT in the subject's HDL [15]. Moreover, lipoprotein lipase (LPL) and hepatic lipase (HL) activities were assessed in post-heparin plasma samples, based on fatty acid release, using a radiolabeled triolein emulsion as substrate and NaCl 1M as LPL inhibitor [16].

### HDL particle size analysis

HDL particle size was measured after chemical precipitation of apo B-containing lipoproteins with polyethylene glycol (PEG) 8000 (400 g/L) in glycine solution 0.2 mol/L, adjusted to pH 10 (Sigma-Aldrich, St. Louis, MO, USA) [17]. Measurements of HDL particle size were made using the Nanotrac Particle Size Analyzer (Microtrac, North Largo Florida, USA) by DLS technique, as described by Lima & Maranhão [18].

### CV risk and carotid atherosclerotic burden estimation

The 10-year risk of coronary fatal or nonfatal myocardial infarction or fatal or nonfatal stroke, and peripheral arterial disease of supposed atherosclerotic origin was estimated by the ASCVD [8]. Measurement of the intima-media thickness (cIMT) of the left and right common carotid arteries was obtained at the far wall and 1 cm from the bifurcation [19] by using a high resolution B-mode carotid ultrasonography (ATL HDI 3500, 6–9 MHz linear transducer, ATL Ultrasound, Bothell, EUA), by a single trained sonographer, according to standardized method. Individual results correspond to the mean of the left and right cIMT in mm.

### Statistical analysis

Distribution of the variables was tested using the Kolmogorov-Smirnov test. Comparative analyses were performed using Kruskal-Wallis for non-normal data, expressed as median (interquartile range), and analysis of variance (ANOVA) for normal data, expressed as mean ± standard deviation. Bonferroni's or Mann-Whitney tests were used for post-hoc analysis. Chi-Square test was used for categorical variables. Analysis of covariance (ANCOVA) adjusted by gender and age was used to compare cIMT between groups. Multivariate ordinal regression models were used to assess the association between cIMT ≥80^th^ percentile (0.90 mm) across increasing levels of lipid parameters. In order to minimize the effect of the differences in magnitudes of the absolute values and make comparable the association between the independent variables and the odds ratios (OR), tertiles of HDL size, HDL-C, LDL-C, non-HDL-C and apo A-I were used as independent variables with the reference group being the lowest tertile. HDL-C was not included in the same model of HDL size due to the presence of colinearity. In the models, we included age, gender, HOMA2S, ethnicity and BMI as covariates because of their known influence on cIMT. A two-sided p-value <0.05 was considered statistically significant. Analyses were performed using SPSS Statistics version 17.0.

## Results

### Clinical characteristics and biochemical data

As shown in Table 1, participants were grouped into tertiles of HDL size (<7.57 nm; 7.57–8.22 nm; and >8.22 nm). Individuals in the 1^st^ tertile presented higher BMI, waist circumference and LAP than those in the 2^nd^ tertile while both were higher than those in the 3^rd^ tertile. Likewise, participants in the 1^st^ tertile had lower levels of HDL-C, apo A-I, HOMA2S, PLTP mass and activity of PON-1 as well as higher levels of insulin, HOMA2B and endogenous LCAT and HL activity as compared to their counterparts. Plasma triglycerides were lower in 3^rd^ tertile group as compared to the others groups. Mean apo B and non-HDL-C were lower in 3^rd^ tertile than in the 2^nd^ tertile. CRP levels and cIMT were also lower in 3^rd^ tertile when compared with others groups.

**Table 1:** Baseline characteristics according to the tertiles of HDL size.

	1^st^ tertile	2^nd^ tertile	3^rd^ tertile	*p*
	(<7.57 nm)	(7.57–8.22 nm)	(>8.22 nm)	
*N*	92	93	99	
HDL size, nm	7.24 (0.36)	7.86 (0.34)	8.51 (0.42)	-
Female, %	49	54	64	0.112
Ethnic group White/non-white, %	77/23	75/25	79/21	0.802
Age, years	49 (13)	51 (14)	52 (13)	0.115
Body mass index, kg/m^2^	24.8±3.1	23.9±2.8	23.4±2.6	0.004^a^
Waist circumference, cm	82±11	78±9	75±9	0.0001^a,b^
Lipid accumulation product - LAP, cm.mmol/L	17 (22)	15 (12)	10 (9)	0.0001^a,c^
Systolic blood pressure, mmHg	120 (20)	120 (15)	120 (20)	0.969
Diastolic blood pressure, mmHg	80 (0)	80 (11)	80 (3)	0.940
HDL-C, mg/dL	39 (22)	63 (25)	75 (13)	0.0001^a,b,c^
Non-HDL-C, mg/dL	124±26	126±27	116±24	0.022^c^
Triglycerides, mg/dL	85 (49)	81 (41)	66 (28)	0.0001^a,c^
LDL-C, mg/dL	106±25	109±24	102±22	0.099
Glucose, mg/dL	87±8	85±10	85±7	0.324
Insulin, uU/mL	5.29 (5.15)	3.70 (3.63)	3.66 (2.95)	0.001^a,b^
HOMA2S, %	169 (148)	239 (279)	232 (250)	0.002^a,b^
HOMA2B, %	81±36	65±28	60±25	0.001^a,b^
Apo A-I, mg/dL	124±29	157±40	178±29	0.0001^a,b,c^
Apo B, mg/dL	82±18	83±19	77±18	0.043^c^
Lipoprotein (a), mg/dL	10.4 (25.0)	17.1 (21.0)	10.7 (23.0)	0.066
GFR, ml/min/1.73m^2^	90 (23)	90 (18)	87 (20)	0.868
CETP, %	14±6	13±6	12±5	0.206
PLTP activity, µmolPC/mL/h	5.74±2.53	5.83±2.49	6.11±2.35	0.564
PLTP mass, mg/L	5.62±1.20	6.54±1.42	6.87±1.23	0.0001^a,b^
PLTP specific activity (µmol/mg/L)	1.07±0.37	0.98±0.30	0.91±0.25	0.019^a^
Hepatic lipase, µmolFFA/mL/h	6.27 (4.98)	4.34 (2.86)	4.12 (4.02)	0.002^a,b^
Lipoprotein lipase, µmolFFA/mL/h	3.29 (3.87)	3.28 (3.79)	4.13 (3.35)	0.408
Exogenous LCAT, nmolCE/mL/h	17±9	17±9	17±8	0.957
Endogenous LCAT, %CE	3.88±1.52	2.86±1.08	2.63±1.10	0.0001^a,b^
PON-1, µmol/min	19 (31)	31 (33)	36 (48)	0.008^a,b^
C-reactive protein, mg/L	1.30 (1.50)	1.06 (1.60)	0.83 (1.30)	0.007^a,c^
PON-1/Apo A-I	0.16 (0.27)	0.20 (0.26)	0.22 (0.26)	0.947
cIMT, mm	0.80 (0.35)	0.71 (0.24)	0.70 (0.19)	0.0001
10-Year ASCV Risk, %	1.25 (2.70)	1.10 (2.60)	0.90 (1.15)	0.156

HDL-C: high-density lipoprotein cholesterol; LDL-C: low-density lipoprotein cholesterol; HOMA2S: homeostasis modeling assessment 2 for insulin sensitivity; HOMA2B: HOMA2 for insulin secretion; Apo: apolipoprotein; GFR: glomerular filtration rate estimated by Modification of Diet in Renal Disease equation; CETP: cholesteryl ester transfer protein; PLTP: phospholipids transfer protein; PC: phosphatidylcholine; FFA: free fatty acids; LCAT: lecithin cholesterol acyltransferase; CE: cholesteryl ester; PON-1: paraoxonase 1; cIMT: carotid intima-media thickness; normal and non-normal data presented as mean ± standard deviation or median (interquartile range) respectively; p values were obtained by ANOVA or Kruskal-Wallis. cIMT comparisons were made by ANCOVA adjusted by age and gender. Significant *a posteriori* differences were obtained by Bonferroni or Mann-Whitney test and were indicated as: a = 1^st^ tertile ≠3^rd^ tertile; b = 1^st^ tertile ≠2^nd^ tertile; and c 2^nd^ tertile ≠3^rd^ tertile.

### HDL size, CV risk and atherosclerosis

The median 10 years ASCVD risk was below 2% in the three groups. There was no significant difference in the mean risk among the HDL size tertiles. Multiple ordinal regressions were performed to estimate the degree of association between the presence of cIMT ≥ the 80^th^ percentile and the independent variables expressed in tertiles: HDL size, HDL-C, LDL-C and non-HDL-C. Displayed in Table 2, the 3 models for each variable: (1) unadjusted; (2) adjusted by age, gender and HOMA2S; (3) adjusted by age, gender, HOMA2S, ethnicity and BMI. HDL particle size >8.22 nm was independently associated with low cIMT both in the unadjusted and adjusted models. We added PON (p = 0.012), CRP (p = 0.028) or LDL-C (p = 0.019) to the third model and the highest tertil of HDL size remained statistically associated with cIMT. HDL-C values (Table 2) and Apo A-I (p = 0.24) were not significantly associated with cIMT. LDL-C≥98 mg/dL and non-HDL-C≥113 mg/dL were both independently associated with higher cIMT in the three models. We did not include waist circumference or LAP due to collinearity between these variables. Be that as it may, exchanging these covariables did not change the statistical significance of the analyses. Insulin was also not included in the models because it is a component of the HOMA2S equation. Since LCAT, HL and PLTP are involved in influencing HDL size these variables were not included in the multivariable models.

**Table 2:** Multivariate ordinal logistic regression analysis using cIMT < and ≥0.90 mm (80^th^ percentile) as dependent variable.

HDL size	<7.57 nm	7.57–8.22 nm	>8.22 nm
	N = 92	N = 93	N = 99
Model 1	Ref group	0.57 (0.23–1.43)	0.40 (0.17–0.97)
		p = 0.229	p = 0.042
Model 2	Ref group	0.57 (0.19–1.71)	0.23 (0.07–0.70)
		p = 0.316	p = 0.010
Model 3	Ref group	0.49 (0.16–1.54)	0.23 (0.07–0.74)
		p = 0.222	p = 0.013

HDL-C	<49 mg/dL	49–71 mg/dL	>71 mg/dL
	N = 91	N = 101	N = 92
Model 1	Ref group	0.73 (0.30–1.82)	0.58 (0.24–1.38)
		p = 0.503	p = 0.216
Model 2	Ref group	0.57 (0.18–1.77)	0.40 (0.13–1.27)
		p = 0.331	p = 0.120
Model 3	Ref group	0.44 (0.13–1.48)	0.43 (0.13–1.38)
		p = 0.187	p = 0.156

LDL-C	<98 mg/dL	98–116 mg/dL	>116 mg/dL
	N = 92	N = 96	N = 96
Model 1	Ref group	4.36 (1.35–14.05)	4.00 (1.25–12.84)
		p = 0.014	p = 0.020
Model 2	Ref group	5.80 (1.37–24.59)	6.07 (1.52–24.25)
		p = 0.017	p = 0.011
Model 3	Ref group	5.76 (1.30–25.58)	6.45 (1.56–26.64)
		p = 0.021	p = 0.010

Non-HDL-C	<113 mg/dL	113–133 mg/dL	>133 mg/dL
	N = 92	N = 96	N = 96
Model 1	Ref group	4.00 (1.25–12.84)	4.36 (1.35–14.05)
		p = 0.020	p = 0.014
Model 2	Ref group	4.45 (1.09–18.24)	5.05 (1.28–19.98)
		p = 0.038	p = 0.021
Model 3	Ref group	3.96 (0.94–16.72)	5.05 (1.26–20.26)
		p = 0.061	p = 0.022

Model 1: unadjusted; Model 2: adjusted by age, gender and HOMA2S; Model 3: age, gender, HOMA2S, ethnicity (white and non-white) and body mass index. Independents variables HDL size, HDL-C, LDL-C e Non-HDL-C divided in tertiles. Results are presented as the odds ratio (95% confidence interval) of cIMT above 80^th^ percentile.

## Discussion

The main finding of the study is that it is not HDL-C levels but HDL particle size that improves the discrimination of individuals with or without increased cIMT among those considered at low CV risk. This association remains significant after adjustment for LDL-C, insulin sensitivity and the presence of traditional CV risk factors.

In NMR studies, plasma concentration of large HDL (9.4–14 nm) has been shown to be inversely associated with CV risk, whereas small HDL (7.3–8.2 nm) has been shown to be positively associated with risk [20]–[22]. Mean HDL size obtained by NMR was inversely associated with cIMT in individuals with familial hypercholesterolaemia and in asymptomatic volunteers [23]. In our study, we enrolled individuals systematically stratified to be at low CV risk by the current ASCVD score. In addition, we used a less costly, easy and consequently more broadly applicable assay for measuring HDL particle size. Consistently, we found that large HDL size (>8.2 nm) is more effective than high HDL-C plasma concentration in discriminating cIMT-estimated atherosclerotic burden in low risk individuals. Furthermore, the observed association was independent of insulin sensitivity, age and LDL-C. In contrast, as expected, both LDL-C and non-HDL-C were strong predictors for increased cIMT.

Although metabolic syndrome as defined by IDF standards was considered an exclusion criterion, individuals in the lower HDL size tertile had higher triglycerides levels, waist circumference and lower insulin sensitivity than their counterparts. Endogenous LCAT and exogenous HL activities were also higher in the 1^st^ tertile, which may have contributed to the reduced HDL size and was possibly favored by the decline in insulin sensitivity [24]–[26]. Likewise, PLTP activity has also been reported to be inversely related to insulin sensitivity and involved in the remodeling of HDL [27]. In agreement with prior studies [28], we found that PLTP mass differed between groups but PLTP activity did not. Consequently, PLTP specific activity, which reflects the relative proportion of active and inactive isoforms, was lower among individuals in the higher tertile of HDL size. Although PLTP effect on HDL size is still controversial, specific PLTP activity seems to be more clearly related to a decreasing effect on particle size [29]. Given this potential modulation of insulin sensitivity in the mechanisms involved in the enlargement of HDL particles, multivariate analyses were performed and confirmed the existence of a direct association between the HDL size and cIMT and plasma CRP. Thus, it is possible that the declined insulin sensitivity and resulting increase in PLTP, LCAT and HL act jointly as a set of stimuli that leads to a reduction in the size of HDL. In turn, this directly and indirectly favors the increase in carotid atherosclerotic burden and systemic inflammatory activity.

In line with this assumption, we found that the overall plasma PON-1 activity was associated with larger HDL size. Besides the effect on cholesterol efflux capacity, this overall antioxidant activity may contribute to the lower association between large HDL and atherosclerotic burden or systemic inflammation. Since small sized HDL particles have been shown to express a higher PON-1 specific activity [30], it is likely that such an increase in overall plasma PON-1 activity in individuals with large HDL size results from the positive association between HDL size and the number of HDL particles. In fact, statistical significance disappeared when the ratio of PON-1 and apo A-I were compared between groups.

In conclusion, the present study indicates that the mean HDL size estimated by DLS constitutes a better predictor for subclinical carotid atherosclerosis than the conventional measurement of plasma HDL-C in individuals classified by the current guidelines as being at low CV risk.
